# Allergy and Household Living Conditions among Adolescents Living near Gold Mine Tailing Dumps in the Gauteng and North West Provinces of South Africa

**DOI:** 10.3390/ijerph19010122

**Published:** 2021-12-23

**Authors:** Abike O. Olajide-Ibiejugba, Vusumuzi Nkosi, Funzani Takalani-Rathogwa, Joyce Shirinde, Janine Wichmann, Robin J. Green, Kuku Voyi

**Affiliations:** 1 School of Health Systems and Public Health, Faculty of Health Sciences, University of Pretoria, Pretoria 0001, South Africa; olawunmiibiejugba@yahoo.co.uk (A.O.O.-I.); joyce.shirinde@up.ac.za (J.S.); janine.wichmann@up.ac.za (J.W.); kuku.voyi@up.ac.za (K.V.); 2 Environment and Health Research Unit, South African Medical Research Council, Johannesburg 2094, South Africa; 3 Department of Environmental Health, Faculty of Health Sciences, Doornfontein Campus, University of Johannesburg, Johannesburg 2094, South Africa; 4 Department of Advanced Nursing Science, Faculty of Health Sciences, University of Venda, Thohoyandou 0950, South Africa; frathogwa@gmail.com; 5 Steve Biko Academic Hospital, Department of Paediatrics and Child Health, Faculty of Health Sciences, University of Pretoria, Pretoria 0001, South Africa; robin.green@up.ac.za

**Keywords:** adolescents, allergy, mine tailing dumps, living conditions, South Africa

## Abstract

This quantitative exploratory baseline study aimed to investigate whether allergy among adolescents was associated with household living conditions, including living near gold mine tailing dumps in South Africa. A questionnaire based on the International Study of Asthma and Allergies was used to collect information on allergy and household risk factors among adolescents (*n* = 5611). A chi-square test was applied to determine the relationship between community (exposed/unexposed) and confounding variables. Crude and adjusted odds ratios (ORs) and 95% confidence intervals (CI) were calculated using univariate and multiple logistic regression analysis (LRA) to estimate the likelihood of having doctor-diagnosed allergies. The overall prevalence of doctor-diagnosed allergies was 25.5%. The exposed communities had a higher prevalence of doctor-diagnosed allergies (26.97%) compared with the unexposed (22.69%) communities. The study found an association between doctor-diagnosed allergy and having fungus in the house, being female, currently having pets in and around the house, residing in the community for more than three years and living in communities located close to gold mine tailing dumps. Actions to implement buffer zones between gold mine tailing dumps and communities would support Sustainable Development Goals 3 (health) and 11 (sustainable cities and communities), while failing to address the current potential identified risk factors may pose a significant public health challenge. Local policymakers should also apply the precautionary principle to protect the health of children, especially with the location of human settlements relative to air pollution sources.

## 1. Introduction

About 20 to 30% of the world’s population have some sort of allergic disease and allergic diseases have now become the most common motive for seeking medical help. Even in its less severe form, it can impact negatively on the health of millions of individuals, reducing the quality of life and work productivity [1,2]. Allergy encompasses conditions that include asthma, allergic rhinitis and has been implicated in adverse drug reactions, atopic dermatitis, occupational diseases, adverse reactions to foods, hypersensitivity reactions of the skin, angioedema and urticaria, insect and bee venom hypersensitivity, conjunctivitis and several diseases of eosinophils and mast cells. [3,4,5,6,7]. They have been described as disorders that are a result of IgE-mediated hypersensitivity, though the underlying mechanism still remains vague [8,9].

Allergies can occur as single entities; however, emerging evidence suggests that co-occurrences of allergies are a common phenomenon [10,11]. Studies estimated that the global prevalence of the co-occurrence of asthma, rhinitis and eczema was nearly 10% higher than what could be expected by probability alone [11,12]. A study in Germany reported the persistence of allergic co-occurrences up to adulthood [13]. Furthermore, people with allergic diseases such as asthma often suffer from other comorbid conditions such as diabetes, obesity, cardiovascular disease, and gastroesophageal disease, which can result in more complex situations and worse outcomes [4,14].

The International Study of Asthma and Allergies in Childhood (ISAAC) globally explored trends in the prevalence of allergic diseases and their risk factors over time in children [15]. Reports based on the ISAAC study showed variations in prevalence between allergies not only within countries but also within the same regions and centres within a country [11,16]. Results from studies conducted in children 13–14 years old show that the 12 month prevalence of eczema, asthma, and rhinitis were between 0.2% and 24.6%, between 3.4% and 31.2%, and between 4.5% and 45.1%, respectively [17,18,19].

An epidemiological study conducted over seven years reported that the prevalence of allergic diseases in Africa are on the rise in adolescents over the years, especially within upper- to middle-income countries such as South Africa [20]. The prevalence of allergic sensitisation is also said to have doubled in recent times, despite this increase, the prevalence rate of 1.2% for developing countries is still lower than the 4.2% reported for developed countries [19,21]. A significant increase in allergic diseases has been reported in various regions of the world at a pace that cannot only be attributed to genetic changes; it is reckoned that environmental factors, including improved hygiene, lifestyle, diet and nutrition, and changes in social structure, increasing industrialisation, and pollution, which are viewed as factors responsible for the rise in allergic conditions in Western populations, play a huge role in this observed global trend [22,23,24,25]. 

Genetic factors have been identified as the greatest risk factor for the development of allergic conditions. In the children of healthy parents, the risk for the development of allergy is between 5–15%, which increases up to 40% when either of the parents are allergic. It could rise to between 60 and 80% if both parents are allergic [24]. This genetic predisposition to developing allergies is also known as atopy. However, the differing prevalence of allergies in persons of similar genetic origin living in different regions of the world may also be attributed to environmental factors [26]. 

The contribution of indoor environmental factors in the triggering and worsening of allergic conditions has been well established [9]. Major components of indoor air pollutants are from combustion sources such as wood, gas, coal and paraffin stoves for cooking, heating and lighting purposes, as well as environmental tobacco smoke [27,28,29,30]. In many low-income and middle-income countries, people rely on solid fuels to heat their homes. Other sources of indoor allergens identified include dust mites, cats, dogs, rodents, cockroaches and a variety of moulds that could exacerbate allergies [4].

Climate change has been shown to contribute to the development of allergic respiratory diseases and asthma by increasing the intensity of indoor and outdoor mould exposure [22,31,32]. Airborne pollen and mould play a significant role in the adverse health outcomes in allergy and asthma [32]. Incessant rain as a result of climate change can increase the number of buildings with damp and mould problems and worsen existing leakages in defective buildings. Studies after hurricane Katrina and Rita in the United States of America revealed moderate to severe fungal growth in houses that were affected [31].

Gold mine tailing dumps are considered to be a major cause of air pollution due to wind and dust that blows, containing complex mixtures of heavy metals and trace elements that are dangerous to human health [33,34]. Studies have shown a high prevalence of asthma symptoms, respiratory diseases, allergic rhinitis, rhinoconjunctivitis and hay fever symptoms among adolescents living close to gold mine tailing dumps in South Africa [33,35,36]. The result of a community-based study in Chile carried out on children living within 2 kilometres of goldmine pits suggest that the closeness of children’s residence to open-pit mines is linked with allergic rhinoconjunctivitis and asthma, with noticeably increased risks in the children living closest to the mines. The results of this study validate the previous study from Latin America which suggests an increased risk for asthma among individuals living within a distance of 2 kilometres to different air pollution sources, including power plants, quarries or petroleum refineries [36,37]. Total suspended particulate (TSP) matter and particles, including an aerodynamic diameter smaller than 10 μm (PM_10_), are the most common air pollutants generated by gold mining activities such as drilling, blasting, sediment loading and unloading, road transport over unpaved roads and losses from exposed overburden gold mine tailing dumps, residual handling plants and exposed pit faces [36]. A study carried out in South Africa suggests that schools located near mine dumps, in comparison with their counterparts that are further away, are exposed to higher concentration levels of outdoor air pollutants such as outdoor PM10 [38]. A research study conducted in Johannesburg showed that environmental standards for PM_10_ are far exceeded in the vicinity of gold mine tailing dumps [39]. Inhalable particulate matter may travel 2 kilometres from the gold mine tailing dumps. An estimated 1.6 million people living in informal or formal settlements on or directly next to gold mine tailing dumps in South Africa may be affected. People living in these settings tend to be historically marginalised and living under the conditions of poverty [40]. 

In South Africa, studies conducted have focused on one or more allergies separately. A systemic review of studies conducted on allergies and asthma have shown that the prevalence of allergic diseases is increasing in Southern Africa [41]. It was also suggested that various factors were responsible for the increase in this situation, especially exposure to certain aeroallergens or other irritants and environmental triggers. This study aimed to investigate whether allergy among adolescents was associated with household living conditions, including living near gold mine tailing dumps among adolescents in the Gauteng and North West provinces of South Africa. To the best of our knowledge, no such study was ever conducted in South Africa or Africa. 

## 2. Materials and Methods

### 2.1. Study Setting and Sample

**Figure 1:**
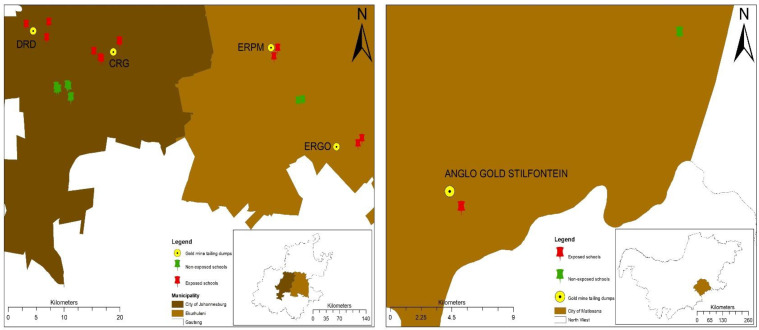
Location of schools selected in the study in relation to proximity to gold mine tailing dumps.

The study participants were 13- to 14-year-olds who resided and attended schools in urban communities located within a 1 to 2 km radius (exposed) or 5 km radius and above (unexposed) of 5 preselected mining dumps in the Gauteng and North West Provinces of South Africa. Eleven communities with similar socioeconomic and demographic profiles were selected, and 13- to 14-year-olds who attended schools in any of these communities were invited to participate in the study. A list of schools in these communities was provided by the Gauteng Department of Education and the North West Provincial Government, and 31 were randomly selected to participate in the study, six of which declined to participate. Altogether, 25 schools participated in the study; 14 schools from exposed communities and 11 schools that were randomly selected from the unexposed communities to match the schools in the exposed communities. Consent forms were delivered to the participating schools 2 weeks before the study, the learners were asked to return them within three days. Of 6500 students eligible to participate in the study, parental consent was given by 5611 learners; the response rate was 91.2%. Table 1 shows the communities selected in the study, and Figure 1 indicates the location of schools selected in the study in relation to proximity to gold mine tailing dumps. These communities were selected because they are situated close to one of the four biggest gold tailings dumps in South Africa and have a high population density around the dumps. 

### 2.2. Study Design

A quantitative exploratory baseline study was conducted between March 2015 and September 2016. There is a paucity of data on the detailed health impact of community proximity to gold mine tailings dumps in South Africa and Africa; the data obtained from this study address the research gap of the lack of allergy studies, environmental exposure and household living conditions in South Africa. This study design was deemed appropriate in answering the question of the prevalence of doctor-diagnosed allergy among adolescents and it allowed for the collection of multiple variables (e.g., environmental factors and demographics data) that are associated with allergy. The learners completed a self-administered questionnaire adapted from the ISAAC phase I protocol (Weiland, 2004). The ISAAC phase I protocol is a multi-centre study involving adolescents in schools from a defined geographical area to understand the aetiology of asthma and allergic diseases. This standardised method allows the description and comparison of the prevalence of asthma and allergy and potential risk factors (e.g., environmental and lifestyle factors) within centres and between countries. This was carried out in the classroom under the supervision of trained data collectors, who had been prepared to avoid explanations that could interfere with participants’ responses. Students not residing in these communities were excluded. Additionally excluded were students and schools who declined to participate or who did not give their approval before the start of the study. The Ethics Committee of the University of Pretoria (Ethics approval number: 303/2014), Gauteng Department of Education and the North West Department of Education approved the study. The study was authorised by the respective school principals and the school governing bodies. Parents or guardians had to give their consent before adolescents could participate in the study. All information was kept confidential.

### 2.3. Questionnaire

The ISAAC Phase I study questionnaire with the standardised core questionnaire on demographics and allergy was used to collect data. Information on potential risk factors was obtained with questions about current living conditions, socioeconomic status, family size, family history of atopic diseases, crowding, pet ownership, active smoking, exposure to environmental tobacco smoke, cooking fuels, damp housing, type of roof, type of floor, type of mattress and number of years lived in the community. The questionnaire was added as a supplement.

### 2.4. Health Outcomes and Potential Risk Factors

The primary health outcome of interest in this study was an allergy, and it was estimated based on affirmative responses to written questions in the modified ISAAC PHASE I questionnaires. The answers to the written questions were self-reported by the study participants. 

The following questions were asked:

1. Do you have any allergies? (Yes/No)

2. Was your allergy diagnosed by a doctor?

### 2.5. Data Analyses

Collected data were entered to Epi Info version 3.5.3 (Centers for Disease Control and Prevention, Atlanta, United States of America) and analysed through STATA 15 (StataCorp, Texas, United States of America). The prevalence of the health outcomes was calculated by dividing the number of study participants who responded affirmatively by the number of the completed questionnaires. A chi-square test was applied to determine the relationship between community (exposed/unexposed) and confounding variables. Crude and adjusted odds ratios (ORs) and 95% confidence intervals (CI) were calculated using univariate and multiple logistic regression analysis (LRA) to estimate the likelihood of having doctor-diagnosed allergies. Missing values were automatically excluded in each LRA model; therefore, each multiple LRA model had a different sample size. To obtain adjusted ORs for the effect of “community (exposed/unexposed)” on the outcome were placed in an initial LRA model. This was followed by the addition of a potential confounder in a stepwise manner starting with the most statistical significant from the univariate analysis. Each time a new potential confounder was added to the model, if the effect estimate between the exposure of interest and the doctor-diagnosed allergy outcome already in the models changed by more than 5%, the additional variable was retained in the final multiple LRA; otherwise, the variable was removed and a different one was added. The most parsimonious multiple LRA models were reported, i.e., those with variables having a *p*-value < 0.05.

## 3. Results

### 3.1. Profile of the Study Participants

Table 2 shows the profile of the population in the study. There were 5611 study participants in the study: 3667 learners were from the exposed and 1944 unexposed communities, respectively. The response rate for the study was 86.3%. In the exposed community 52.33% were female, 25.31% lived in the area for more than 3 years, 86.09% lived in formal dwellings (a house built by a professional builder and made of bricks with building plans approved by the local municipality), 7.80% lived in a house with damp walls, 6.14% lived in a house with fungus, 44.91% had pets in and around the house, 4.77% used a wood stove while 8.81% used a coal stove as a source of energy in the house, 8.37% used paraffin and 73.55% electricity, 38.01% were exposed to environmental tobacco smoke and 4.61% smoked cigarettes.

### 3.2. Prevalence Doctor-Diagnosed Allergy

The overall prevalence of self-reported allergy for the total study population was 40.36% and doctor-diagnosed allergy was 25.50%. The exposed communities had a higher prevalence of self-reported allergy 41.40% and doctor-diagnosed allergy of 26.97% compared to the unexposed with self-reported allergy of 40.36% and doctor-diagnosed allergy of 22.69% communities, respectively, the results are shown in Table 3. 

### 3.3. Univariate and Multiple LRA of Doctor-Diagnosed Allergy and Household Living Conditions

The result from the univariate and multiple LRA (Table 4) shows that living in exposed communities (OR = 1.23; 95% CI: 1.07–1.61) and the house having fungus (OR = 1.31; 95% CI: 1.00–1.63) were potential risk factors for allergy among adolescents. However, a significant protective effect was observed or having pets in and around the house (OR = 0.89; 95% CI: 0.84–0.95), being a female (OR = 0.47; 95% CI: 0.41–0.55) living in the community for the duration of more than 3 years (OR = 0.78; 95% CI: 0.68–0.90). 

## 4. Discussion

The purpose of the study was to investigate whether allergy among the adolescents was associated with household living conditions, including living near gold mine tailing dumps in the Gauteng and North West Provinces of South Africa. The main findings of the study show that the overall prevalence of self-reported and doctor-diagnosed allergy was higher in the exposed (41.40%; 26.97%) compared to unexposed (38.78%; 22.69%) communities, respectively. 

The difference observed in the prevalence of allergy between the exposed and unexposed communities is not very large. This may be attributable to the distance between the gold mine tailings dumps and the two communities. The wind direction and speed may also be a contributing factor as dust particles from the mine may be transported by the wind and deposited much further away from the gold tailings mine dumps. It is well known that smaller dust particles and other pollutants may travel long distances and be deposited in communities much further away from the original source. 

Zar et al. [20] argued that the prevalence of allergic diseases such as asthma, allergic rhinitis, and atopic eczema are increasing in developing countries as well as in South Africa. The rise in the prevalence is attributed to risk factors such as diet, exposure to air pollution, inactivity, and exposure to indoor allergens, amongst others. In the current study, living in exposed communities significantly increased the prevalence of allergy. This could be because mine dumps are a major cause of air pollution due to wind and dust that blows, containing complex mixtures of heavy metals and trace elements that are dangerous to human health [34,42]. In other similar studies conducted in children, exposure to air pollution from trucks was significantly associated with hay fever, allergic rhinitis and rhinoconjunctivitis [33,35]. A significant association between doctor-diagnosed allergy and living in exposed communities was observed. These findings are similar to that of a study that investigated the prevalence of asthma, wheeze and rhinoconjunctivitis, which are considered symptoms of asthma among exposed and unexposed communities, in which the results suggest that the prevalence of wheeze and rhinoconjunctivitis was high in exposed communities when compared to unexposed communities. In that study, the prevalence of asthma, wheeze and rhinoconjunctivitis were 17.5%, 18.5% and 29.1%, respectively [42]. It is interesting to note that, as in the current study, a high prevalence of allergies was observed in exposed communities. Another study conducted in Polokwane reported a prevalence of current wheeze, current severe wheeze, eczema and rhinoconjunctivitis as 11%, 6%, 8% and 7.3%, respectively [43].

Being female was a significant protective factor against allergy. These results are contradictory to the findings of a study that proved that both endogenous and exogenous sex steroid hormones contribute to the occurrence of allergies such as asthma and wheeze in young women [44,45]. There is a growing body of evidence that hormonal factors, especially estrogen and progesterone, affect the occurrence of allergies [44]. In other epidemiological studies, there was a significant relationship between hormonal fluctuations during menstruation and the exacerbation of asthma [46,47]. Another study also reported that although allergic rhinitis and airway hyperresponsiveness is much higher in boys, it is more severe in girls [45]. This result could have been due to chance as it is known that being female is a predisposing factor to diseases not only respiratory in nature but also cancers, and this is mainly due to hormones.

In the current study, living in the community for a duration of more than 3 years was potentially significantly protective from allergy. This result is contrary to other studies conducted and could have been entirely due to chance, because the communities included in the study are based 1–5 km away from gold mine tailings dumps. Gold mine tailing dumps are a major source of particulate matter pollution and other pollutants, which are transported by wind to nearby communities [33,48]. All these communities have been exposed to air pollution, although some to a lesser extent. Air pollution exposure for extended periods should be associated with allergy and other diseases. Particulate matter and other air pollutants have detrimental effects on the respiratory system and studies conducted have shown that exposure to particulate matter is associated with respiratory diseases, such as chronic bronchitis, emphysema and airflow obstruction [49,50]. 

Having fungus in the house significantly increased the prevalence of allergy among participants. This agrees with evidence from the literature that suggests that other sources of indoor allergens include dust mites, cats, dogs, rodents, cockroaches and a variety of moulds that could exacerbate allergies [4]. Furthermore, according to Jenerowicz et al. [51] some of the most extensively studied environmental factors influencing allergy are airborne allergens: dust mites, pollens, fungi and animal dander. Some studies have suggested that having damp walls and carpets in the house was associated with allergies, but in another study, an association between asthma among children and dampness was not observed [52]. There is a high prevalence of fungal sensitization among Africans with asthma, but there remains a paucity of data on the epidemiology and associated complications [53]. There is an urgent need for national epidemiological studies to estimate the actual burden of fungal asthma in Africa [53]. Therefore, this study adds to the paucity of data on household living conditions and indoor fungus. 

However, in this study, having pets in and around the house was a potential significant protective factor. This agrees with emerging evidence that suggests an inverse association between domestic cats, dog ownership and allergic disease. The protective effect of cat allergen exposure to allergy could be explained by the adjuvant effect of the peptides [54]. Evidence from epidemiological studies suggests that living with a dog or a cat was an important risk factor to allergy and was also associated with a higher risk of wheeze [51]. Another study found that the presence of at least one furred pet at home is associated with an increased prevalence of allergic symptoms [55]. Certain limitations should be taken into account when interpreting the results. Firstly, the study cannot produce any evidence of causality. Secondly, no quantitative air pollution exposure assessment was conducted. Thirdly, no allergy tests were conducted during the study. Thirdly, the differential participation rate between the exposed and unexposed communities is of concern and may well have introduced response bias, which is likely to overestimate the prevalence calculated and also bias the association in either direction. Lastly, data on natural ventilation were not collected. The strength of this study is that an international validated ISAAC questionnaire was used to study allergy and household living conditions, with a high sample size. The temporal relation allergy and potential risk were measured at the same time. The results of this study can serve as a basis for further detailed epidemiological studies for communities located near gold mine tailings in South Africa, e.g., a planned birth cohort study. 

## 5. Conclusions

The study found an association between doctor-diagnosed allergy and having fungus in the house, being female, currently having pets in and around the house, residing in the community for more than three years and living in communities located close to gold mine tailing dumps. The results of the study are similar to other studies in the literature, which reported an association between doctor-diagnosed allergy and exposure to allergens such as the presence of fungus in the house. Although being female was found to be a protective factor, this phenomenon is still not clearly understood and warrants further investigation. Having pets in and around the house was found to be associated with doctor-diagnosed allergy; this finding is supported by many other studies in the literature, although some researchers have observed the opposite, where researchers argue that children who grow up with animals around the house may build an immunological response, serving as a protective factor. Residing in the community for more than three years and living in close proximity to the gold mine tailings dumps were also associated with doctor-diagnosed allergy. This finding is important and supports the evidence of the many health effects experienced by communities living in close proximity to gold mine tailings dumps. The results of the study add to the number of limited studies carried out in developing countries such as South Africa, which focus on the health impacts in communities located close to gold mine tailing dumps. Most of the mining companies left these communities decades ago, leaving the gold mine tailing dumps unattended or not rehabilitated. The baseline data from this study will serve as a benchmark for future epidemiological studies and exposure assessment studies to build more evidence on the health effects associated with living close to gold mine tailing dumps. Actions to implement buffer zones between gold mine tailings and communities would support Sustainable Development Goals 3 (health) and 11 (sustainable cities and communities), while failing to address the current identified potential risk factors may pose a significant public health challenge. Local policymakers should also apply the precautionary principle to protect the health of children, especially with the location of human settlements relative to air pollution sources.

## Figures and Tables

**Table 1:** Thirteen communities selected in the study located in Gauteng and North West Provinces of South Africa.

Gold Mine Tailing Dumps	Province	Exposed Community ^a^	Unexposed Community ^b^
Crown Gold Recoveries (CGR)	Gauteng	Riverlea, Deepkloof, and Noordgesig	Pimville, Dlamini and Eldorado
East Rand Proprietary Mines (ERPM)	Gauteng	Reigerpark	Windmill Park
Ergo	Gauteng	Geluksdal	Windmill Park
Anglo Gold Stilfontein	North West	Stilfontein	Jouberton

^a^ Exposed: communities located 1–2 km from mine dumps. ^b^ Unexposed: communities located 5 km or more from mine dumps.

**Table 2:** Household characteristics of study participants (*n* = 5611).

Characteristic	Community Type			^c^*p*-Value
	^a^ Exposed (*n* = 3667)	^b^ Unexposed (*n* = 1944)	Total (*n* = 5611)	
Gender				0.129
Male	1748 (47.67)	968 (49.79)	2716 (48.40)	
Female	1919 (52.33)	976 (50.21)	2895 (51.60)	
Number of years in community				0.002
>3 years	2739 (74.69)	1525 (78.45)	4264 (75.99)	
≤3 years	928 (25.31)	419 (21.55)	1347 (24.01)	
Type of dwelling				<0.001
* Formal	3157 (86.09)	1574 (80.97)	4731 (84.32)	
# Informal	510 (13.91)	370 (19.03)	880 (15.68)	
Damp walls in the house				0.010
Yes	286 (7.80)	191 (9.83)	477 (8.50)	
No	3381 (92.20)	1753 (90.17)	5134 (91.50)	
Fungus in the house				<0.001
Yes	225 (6.14)	185 (9.52)	410 (7.31)	
No	3441 (93.84)	1759 (90.48)	5200 (92.67)	
Missing	1 (0.03)	0 (0.00)	1 (0.02)	
Currently have pets in and around the house				0.328
Yes	1647 (44.91)	914 (47.02)	2561 (45.64)	
No	2020 (55.09)	1030 (52.98)	3050 (54.36)	
Use of wood stove				0.055
Yes	175 (4.77)	122 (6.28)	297 (5.29)	
No	3492 (95.23)	1822 (93.72)	5314 (94.71)	
Use of coal stove				0.541
Yes	323 (8.81)	155 (7.97)	478 (8.52)	
No	3344 (91.19)	1789 (92.03)	5133 (91.48)	
Use of paraffin				0.591
Yes	306 (8.37)	185 (9.52)	491 (8.75)	
No	3360 (91.63)	1759 (90.48)	5119 (91.23)	
Missing	1 (0.03)	0 (0.00)	1 (0.02)	
Use of electricity				0.005
Yes	2697 (73.55)	1351 (69.50)	4048 (72.14)	
No	970 (26.45)	593 (30.50)	1563 (27.86)	
Exposure to ETS at home in the past 30 days				0.436
Yes	1394 (38.01)	735 (37.81)	2129 (37.94)	
No	2273 (61.93)	1209 (62.19)	3482 (62.06)	
Do you smoke?				<0.001
Yes	169 (4.61)	89 (4.58)	258 (4.60)	
No	3498 (95.39)	1855 (95.42)	5353 (95.40)	

Figures in parentheses are percentages. ^a^ Exposed: communities located 1–2 km from mine dumps. ^b^ Unexposed: communities located 5 km or more from mine dumps. ^c^ *p*-values of the Chi-squared test. * A house built by a professional builder and made of bricks with building plans approved by the local municipality. # A house made of corrugated ion, wood and mud structures without building plans and not approved by the local municipality.

**Table 3:** Prevalence of allergy among the adolescents (*n* = 5611).

		Community		Total	^c^*p*-Value
		^a^ Exposed	^b^ Unexposed		
Do you have any allergy?					
	Yes	1518 (41.40)	746 (38.37)	2265 (40.36)	0.043
	No	2149 (58.60)	1198 (61.63)	3346(59.64)	
	Total	3667(65.35)	1944 (34.64)	5611 (100)	
Was the allergy diagnosed by the doctor?					
	Yes	989 (26.97)	411 (22.69)	1431 (25.50)	<0.001
	No	2678 (73.03)	1503 (77.31)	4181 (74.50)	
	Total	3667 (64.35)	1944 (34.64)	5611 (100)	

Figures in parentheses are percentages. ^a^ Exposed: communities located 1–2 km from mine dumps. ^b^ Unexposed: communities located 5 km or more from mine dumps. ^c^ *p*-values of the Chi-squared test.

**Table 4:** Crude and adjusted odds ratios of the doctor-diagnosed allergy and household living conditions.

Characteristics	Crude ORs	95% CI	*p*-Value	Adjusted ORs	95% CI	*p*-Value
Community type						
Unexposed	1	1	1	1	1	1
Exposed	1.26	1.10–1.43	<0.001	1.23	1.07–1.61	0.001
Gender						
Male	1	1	1	1	1	1
Female	0.50	0.44–0.56	<0.001	0.47	0.41–0.55	<0.001
Number of years in the community						
>3 years	1	1	1	1	1	1
≤3 years	0.81	0.71–0.93	0.003	0.78	0.68–0.90	0.001
Type of dwelling						
Formal	1	1	1	1	1	1
Informal	1.27	1.08–1.48	0.004	0.84	0.71–1.60	0.101
Damp walls in the house						
No	1	1	1	1	1	1
Yes	1.24	1.01–1.52	0.044	1.18	0.95–1.46	0.132
Fungus in the house						
No	1	1	1	1	1	1
Yes	1.26	1.01–1.57	0.004	1.31	1.00–1.63	0.049
Currently have pets in and around the house						
No	1	1	1	1	1	1
Yes	0.93	0.88–0.98	0.039	0.89	0.84–0.95	0.001
Use of coal stove						
No	1	1	1	-	-	-
Yes	1.21	0.98–1.48	0.077	-	-	-
Use of wood stove						
No	1	1	1	-	-	-
Yes	1.06	0.82–1.38	0.655	-	-	-
Use of paraffin stove						
No	1	1	1	-	-	-
Yes	0.99	0.80–1.21	0.895	-	-	-
Use electricity						
Yes	1	1	1	-	-	-
No	0.95	0.83–1.08	0.434	-	-	-
Exposure to ETS at home in the past 30 days						
No	1	1	1	-	-	-
Yes	0.94	0.83–1.07	0.348	-	-	-
Do you smoke cigarette?						
No	1	1	1	1	1	1
Yes	0.78	0.57–1.06	0.108	0.85	0.62–1.16	0.312

Model adjusted for community type, gender, number of years living in the community, type of dwelling, damp walls in the house, fungus in the house, currently having pets in and around the house and smoking cigarettes.

## Data Availability

We did not receive ethics approval to share raw field data publicly. The data belong to the University of Pretoria (UP). The raw data analysed in the current study are available from UP on reasonable request.
